# Early Pleistocene climate in western arid central Asia inferred from loess-palaeosol sequences

**DOI:** 10.1038/srep20560

**Published:** 2016-02-03

**Authors:** Xin Wang, Haitao Wei, Mehdi Taheri, Farhad Khormali, Guzel Danukalova, Fahu Chen

**Affiliations:** 1 Key Laboratory of Western China’s Environmental Systems (Ministry of Education), College of Earth and Environmental Sciences, Lanzhou University, Lanzhou 730000, China; 2 Department of Soil Science, Gorgan University of Agricultural Sciences and Natural Resources, Gorgan 49138-15739, Iran; 3 Institute of Geology Ufimian scientific Centre, Russian Academy of Sciences, Ufa 450077, Russia; and Kazan Federal University, Kazan 420008, Russia

## Abstract

Arid central Asia (ACA) is one of the most arid regions in the mid-latitudes and one of the main potential dust sources for the northern hemisphere. The lack of *in situ* early Pleistocene loess/dust records from ACA hinders our comprehensive understanding of the spatio-temporal record of aeolian loess accumulation and long term climatic changes in Asia as a whole. Here, we report the results of sedimentological, chronological and climatic studies of early Pleistocene loess-palaeosol sequences (LPS) from the northeastern Iranian Golestan Province (NIGP) in the western part of ACA. Our results reveal that: 1) Accumulation of loess on the NIGP commenced at ~2.4–1.8 Ma, making it the oldest loess known so far in western ACA; 2) the climate during the early Pleistocene in the NIGP was semi-arid, but wetter, warmer, and less windy than during the late Pleistocene and present interglacial; 3) orbital-scale palaeoclimatic changes in ACA during the early Pleistoceneare in-phase with those of monsoonal Asia, a relationship which was probably related to the growth and decay of northern hemisphere ice sheets.

The Asian interior constitutes the largest mid-latitude arid and semi-arid zone on Earth. Distributed along the main transport pathways of the zonal Westerlies, huge amounts of dust generated in this region were transported by the Westerlies to North China^1^,^2^, the North Pacific Ocean^3^, and as far as the Atlantic region^4^. The mobilization, transportation, and deposition of Asian dust played an important role in global mineral dust cycles, and had a prolonged and profound impact on global climate changes via direct effects on Earth’s radiative balance as well as by various indirect effects^5^. Thus, reconstruction of the spatio-temporal history of aeolian dust and palaeoclimate in ACA is important for understanding the forcing mechanisms of palaeoclimatic changes in the Asian interior on various time-scales and for predicting future regional climate changes in semi-arid and arid regions under global warming.

According to the dominant wind patterns, the mid-latitudes of the Asian continent can be divided into Westerly-dominated ACA and Monsoon-dominated Asia^6^ (Fig. 1a). The former stretches roughly west-east from the Caspian Sea in northern Iran to the regions along the Tengger Desert in northwestern China^7^. The precipitation in these areas is mainly influenced by the location and intensity of the zonal Westerlies, while the dust is mainly transported by regional westerly and northwest-northeasterly winds. The latter are mainly restricted to eastern China, where the precipitation is influenced by the East Asian summer monsoon (EASM) and the Indian summer Asian monsoon (ISA), while the dust is mainly transported by the East Asian winter monsoon (EAWM) (Fig. 1a). Various studies indicate that the effective moisture history in ACA exhibited an out-of-phase or anti-phased relationship with monsoonal Asia on inter-annual to multi-millennial time scales during the Holocene^6^,^7^,^8^,^9^. However, orbital-scale palaeoclimatic changes in ACA, as well their relationship with monsoon Asia during the Pleistocene, remain unclear.

Loess deposits in the mid-latitudes of Asia are unique long-term terrestrial archives which can be used to reconstruct changes in continental climate on orbital time-scales^1^,^10^,^11^. Previous palaeoclimatic investigations of loess deposits mainly focused on the well-known LPS and the underlying red clay formation on the Chinese Loess Plateau (Fig. 1a). During the past three decades, numerous studies have extended this record back to the early Pleistocene and even to the late Oligocene^11^,^12^,^13^,^14^,^15^. These studies demonstrate that colder and drier climates occurred during glacials, and warmer and wetter climates during interglacials, in monsoonal Asia during the Pleistocene^10^,^12^,^16^, and that this relationship was triggered mainly by the Northern Hemisphere glaciations via their impact on the EASM^10^,^17^,^18^. However, the relationship between loess accumulation and long-term climatic history in ACA remains unclear, largely due to the lack of known LPS in this remote region.

The loess cover in ACA is developed as a belt along the southern and southeastern margins of the Karakum, Kyzylkum, Muyunkum, Gurbantunggut, and Taklimakan deserts^19^ (Fig. 1a). Most loess records reported from ACA cover a time span from the middle-late Pleistocene to the present^20^,^21^,^22^,^23^,^24^. The only known lower Pleistocene loess, with a basal age of ~2-2.5 Ma, is the Karamaidan section from south Tajikistan^19^. Thus, the work carried out so far raises the following questions: Are there any older loess deposits in ACA than have heretofore been discovered? What is the spatial distribution of the lower Pleistocene loess in ACA? What are the characteristics of the early Pleistocene climate in ACA and how they did they vary on an orbital time scale? What is the relationship of climatic changes in the region during the Pleistocene with those of monsoonal Asia?

Here, we attempt to address these questions via an analysis of the stratigraphy, origin, chronology, and palaeoclimatic proxies of the red-coloured sediments underlying middle-to-upper Pleistocene loess successions in the NIGP (Fig. 1b) (see *SI* text for more detailed information on the geological setting).

## Results

### Stratigraphy

**Figure 1:**
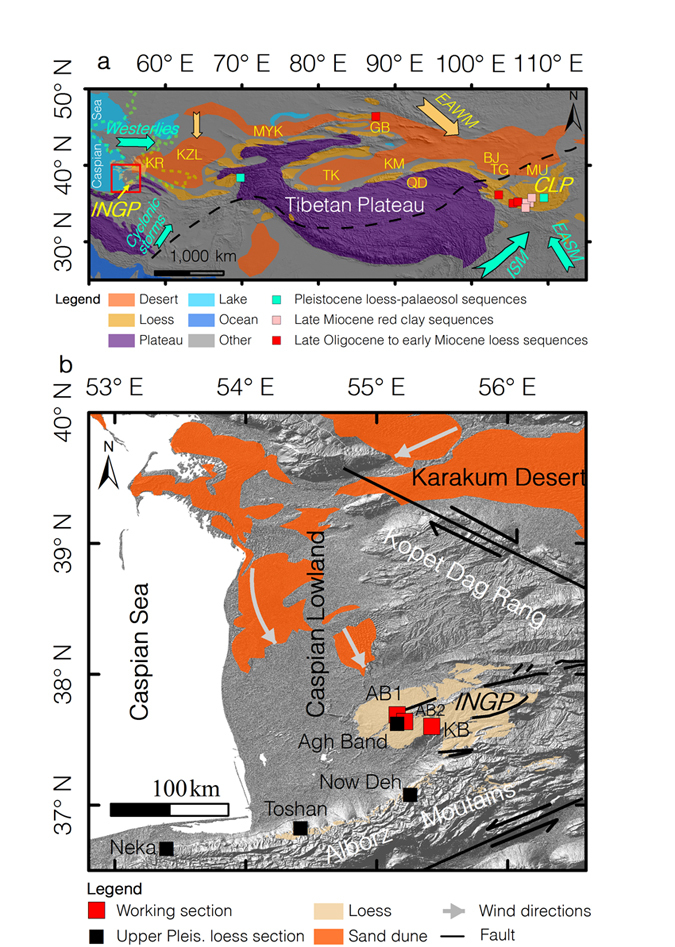
Location of the studied sections. (**a**) Schematic map showing loess and desert distribution in mid-latitude Asia (modified from ref. 19). The green and yellow arrows indicate the major moisture sources and dust- transporting winds for the loess regions. The black dashed line indicates the boundary between ACA and monsoon-dominated Asia^6^. The green dashed line indicates the coastline of Paratethys during the Akchagylian stage (after ref. 43). Abbreviations for deserts: KR - Karakum, KZL - Kyzylkum, MYK - Muyunkum, GB - Gurbantunggut, TK - Taklimakan, KM - Kumtag, QD - Qaidam, BJ - Badain Jaran, TG - Tengger, MU - Mu Us; (**b**) Schematic map showing the location of studied sections, loess distribution, tectonic regime, and the dominant near-surfacewind directions around the NIGP. The data for drawing the distribution of loess in northern Iran comes from the Golestan Natural Resources and Watershed Management Central Office. The sand dune distributions were redrawn from ref. 55. The major faults in northern Iran were redrawn from ref. 56. The map was generated by the software of ESRI ArcGIS v9.3 using the SRTM DEM data (http://srtm.csi.cgiar.org).

**Figure 2:**
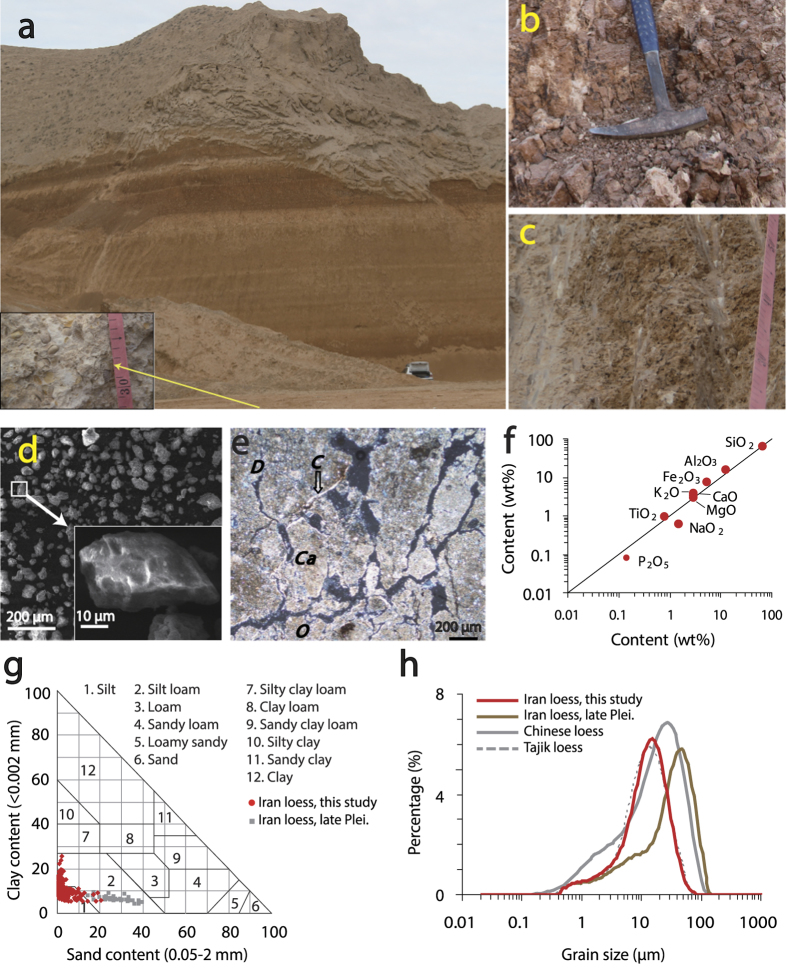
Lithology and sedimentology of the reddish loess studied in the AB1 section. (**a–c**) Photograph showing the lithology of the studied section; (**d**) SEM image showing the angular morphology of quartz grains; (**e**) thin section showing pedogenic features within palaeosols: *D* - Calcite depletion zones dominated by speckled b-fabric, *C* - Clay coatings along channels, *Ca* - Calcitic crystallitic b-fabric, *O* - dominance of Fe/Mn oxides as coatings in the matrix. (**f**) comparison of the major element compositions between the lower Pleistocene loess and the overlying upper Pleistocene loess in the AB1 section; (**g**) comparison of the textural classification of the red-coloured sediments and the overlying upper Pleistocene loess from the AB1 section (grey squares) (the classification was made using the computer program described in ref. 57); (**h**) grain-size distribution of representative samples and its comparison with those of typical loess from the overlying upper Pleistocene loess (this study), south Tajikistan^28^, and the CLP^29^. It should be noted that the laser diffraction method may potentially underestimate the content of the clay fraction. The figure was generated using Adobe Illustrator.

Red-coloured non-marine sediments, with a thickness of about 20 m, are widely distributed in the NIGP (Fig. 1b, Fig. S1). These red beds unconformably underlie the upper Pleistocene loess successions^23^ and conformably overlie the late Cenozoic limestone sequences that include abundant mollusc shells (Fig. 2a). The investigated sections generally dip 5–10° NNW, and are dominated by fine-grained silts with a massive structure. The red-coloured sequences consist of alternations of reddish-yellow (10 YR 6/6) loess-like layers and brownish-red (7.5 YR 3/6) palaeosols. The palaeosols (Fig. 2b) are strongly developed and contain large (up to ~20 cm diameter) carbonate nodules, as well as gypsum, and are commonly underlain by carbonate-rich horizons. In contrast, the loess-like horizons have a massive structure and contain small carbonate nodules (<2 cm), wormholes and iron-manganese coatings (Fig. 2c). These sedimentary characteristics are similar to those of the lower Pleistocene loess successions from the CLP, an equivalent of the Wucheng formation^1^,^16^.

### Sedimentology

The quartz grains within the loess-like horizons are well-sorted and have irregular shapes with sharp edges and dish-shaped concavities (Fig. 2d), suggesting prolonged aeolian transport and thus a wind-blown origin^11^,^25^. Microscope analysis of thin sections from the palaeosol layers revealed an overall well-separated soil microstructure (Fig. 2e). The dominance of channel voids, speckled and partly- calcitic crystallitic b-fabric, clay coatings, and iron-manganese coatings indicated that the soil is moderately- to well-developed^26^. The geochemical composition of the red-colored sedimentsis similar to the overlying upper Pleistocene loess (Fig. 2f), suggesting a similar origin. This geochemical composition is comparable to the average composition of the upper continental crust, indicating that the sediments were derived from well-mixed sedimentary material that has undergone numerous stages of recycling within the upper crust, and is interpreted as a basic feature of loess deposits^27^. Grain-size data of 510 samples from the AB1, AB2, and KB sections reveal that the red beds are dominated by fine-grained silts (Fig. 2g), with an average silt content of 86.5% (the average clay and sand contents are 10.9% and 2.6%, respectively). This silty texture is similar to, but somewhat finer than, that of the overlying upper Pleistocene loess deposits (Fig. 2g) and Chinese loess^1^. The lack of coarse grains is widespread and the thickness of the strata and the similarity of the soil texture to typical loess from the mid-latitudes of Asia suggest that the red-coloured sediments are aeolian in origin. Grain-size distributions of the red-colored sediments are typically bimodal with a well-sorted coarse component (10-20 μm) and a minor ultra-fine component (<2 μm) (Fig. 2h). This grain-size distribution is comparable to that of the overlying upper Pleistocene loess in the NIGP, the Pleistocene loess from south Tajikistan^28^ and the CLP^29^, and is interpreted as characteristic of loess deposits^28^,^29^. In summary, all of the aforementioned sedimentological and lithological evidence indicates that the widely distributed red-coloured silt-dominated sediments underlying the upper Pleistocene loess successions in the NIGP are aeolian in origin.

### Chronology

**Figure 3:**
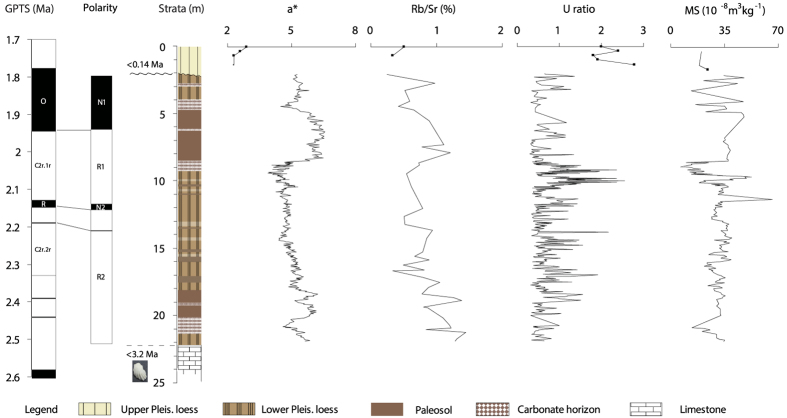
Chronological framework based on magnetostratigraphy and variation of multi-proxy indices of the AB1 section. The depth of 0 m was assigned to the lower part of the overlying upper Pleistocene loess strata, 2 m above the top of the red beds. ‘O’ and ‘R’ in the GPTS^30^ represent the Olduvai and Reunion subchrons, respectively.

**Figure 4:**
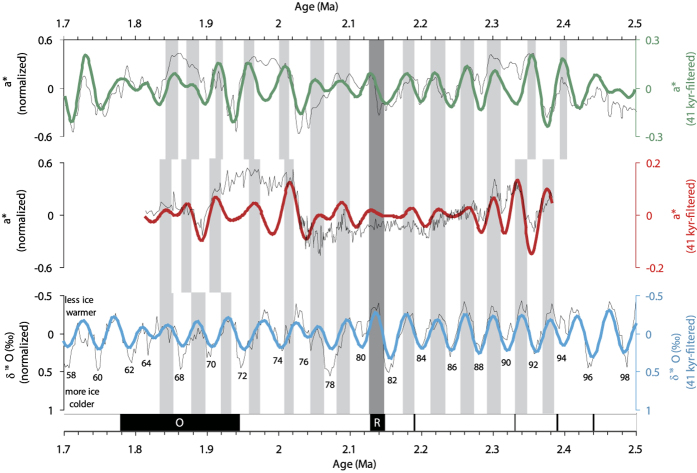
Comparison of redness records from the AB1 section in the NIGP and the Lingtai section^31^ on the CLP, and the LR04 benthic δ^18^O stacked curves^32^. The 41-kyr periodicity band (bold coloured lines) was extracted using the computer program developed by Paillard^58^; band-pass filters with a central frequency of 0.02439 ka^−1^ and bandwidth of 0.01 ka^−1^ were used. All of the records and filtered signals are normalized.

Mollusc species collected from the underlying limestone sequences indicate an Aktschagylian age of the deposits, suggesting that the limestone and the overlying red beds must be younger than 3.2 Ma (Fig. S2, and see *SI* Text for more information). The palaeomagnetic polarity of the red-colored strata in the AB1 section consists of two normal and two reversed intervals, separated by a single normally-magnetized sample at 10.7 m (Fig. 3, Fig. S3-S5 and see *SI* Text for more information). With the above biostratigraphic age constraints, N1 and N2 are logically correlated to the Olduvai (O) subchron (C2n) and Reunion (R) subchron (C2n.1n), and the normal event at 10.7 m is correlated to the geomagnetic excursion occurring at 2.19 Ma^30^ (Fig. 3, see *SI* Text for more information). Based on this correlation, the sediment accumulation rates within the R1 and N2 intervals are ca. 3.03 cm/kyr and 2.29 cm/kyr, respectively. By using the sediment accumulation rate from the neighboring stratum, the ages of the base and top of the red-colored sediments within the AB1 section are ~2.38 and ~1.81 Ma, respectively. The reliability of the palaeomagnetic chronology is supported by the coherence of the redness records from the AB1 section and the Lingtai section on the CLP^31^ with the LR04 stacked benthic δ^18^O (%) records^32^ (Fig. 4, and see *SI* Text for more information).

### Palaeoclimatic records

Colour is the most easily visualized property of sediments and red and yellow are the dominant colours of the LPS in the mid-latitudes of Asia. Reddish colours are ascribed to the presence of the mineral hematite (a-Fe_2_O_3_), which forms under a hot and dry climate, while yellowish colours are commonly associated with goethite (a-FeOOH) which forms in a cool and more humid climate^33^. Thus, redness (a*) can be used as a sensitive and reliable proxy index for regional moisture and/or temperature changes in the mid-latitudes of Asia^20^,^31^,^34^. Redness in the lower Pleistocene reddish LPS in the NIGP is generally higher in the palaeosols (ca. 5.5) and lower in the loess horizons (ca. 4.9), suggesting a higher temperature and/or more humid climate when the palaeosols developed. Redness exhibits an overall decreasing trend within the reddish loess successions, reflecting long-term cooling and enhanced aridity in the NIGP from ~2.4 to 1.8 Ma. The average redness (a*) in the lower Pleistocene loess successions (ca. 5.1) is significantly higher than in the overlying upper Pleistocene loess (ca. 2.5) and modern loess (ca. 2.8) (Fig. 3), suggesting that the early Pleistocene climate in NIGP was warmer and/or more humid than the late Pleistocene and modern climate.

Rb/Sr ratio is an effective proxy index for regional weathering intensity for LPS^35^,^36^. Rb is an immobile element and is always incorporated in K-containing minerals which are relatively resistant during chemical weathering. In contrast, Sr is chemically highly reactive and is readily leached out during pedogenesis due to the loss of CaCO_3_ and other Ca-bearing minerals^37^. Due to the differential mobility of these two trace elements during chemical weathering, Rb is generally enriched and Sr is depleted in the weathering profiles of various parent rocks^37^. As leaching processes during pedogenesis mainly depend on mean precipitation, the Rb/Sr ratiois are sensitive to the amount of palaeo-precipitation^35^. The Rb/Sr ratio exhibits a decreasing trend within the reddish LPS (Fig. 3), reflecting the long-term intensification of aridification in the NIGP from ~2.4 to 1.8 Ma. Across the AB1 section, the Rb/Sr ratio is systematically higher in the lower Pleistocene LPS (ca. 0.92) than in the upper Pleistocene loess and modern loess (ca. 0.42) (Fig. 3), suggesting stronger chemical weathering and thus a wetter climate in the NIGP during the early Pleistocene.

The grain size of loess deposits is a proxy for palaeo-wind strength and/or the distance between the studied sites and the sources^17^,^20^,^28^,^29^,^38^. The proportions of different size fractions potentially provide detailed information on palaeoclimatic changes. U ratio, representing the ratio of coarse (44-16 μm) to fine (16-5 μm) fractions in loess samples^39^, has been widely used as a proxy for wind strength in LPS^21^,^39^. A higher U ratio reflects the dominance of coarse particles in a sample and indicates relatively strong winds, and *vice versa*. In the AB1 section, U ratio is higher in the loess strata (ca. 0.76) than in the strongly-developed palaeosols (ca. 0.69), suggesting a greater wind strength during the periods when the loess accumulated. The average U ratio in the lower Pleistocene LPS in the NIGP is 0.75, significantly lower than that of the overlying upper Pleistocene loess and modern loess (ca. 1.99) (Fig. 3). This suggests that the near-surface wind regime was less vigorous than during the late Pleistocene.

The magnetic susceptibility of loess deposits is a function of the types, concentration, and grain size of the constituent magnetic minerals^40^ and has been widely used in palaeo-climatic reconstruction^10^,^11^,^12^. More detailed characterization of the magnetic properties of the Iranian LPS is underway; however, here we note that the magnetic susceptibility in the lower Pleistocene LPS is higher in the well-developed palaeosols than in the adjacent loess strata. This probably suggests that the magnetic enhancement in the Iranian LPS is mainly related to the formation of ferromagnetic minerals during post-depositional pedogenesis. The magnetic susceptibility is systematically higher in the lower Pleistocene LPS (except in some carbonate-rich layers) than in the overlying upper Pleistocene loess succession. This long-term trend suggests that the early Pleistocene climate in the NIGP was warmer and/or more humid than the late Pleistocene and modern climate.

## Discussion

Sedimentological evidence confirms that the widespread red-coloured sediments underlying the upper Pleistocene loess in the NIGP are aeolian in origin. Moreover, since they are located downwind and on the periphery of large deserts, and are far from areas affected by major glaciations (Fig. 1b), the loess deposits on the NIGP are undoubtedly “*desert”* loess^41^. Several lines of evidence suggest that the Karakum Desert and the arid Caspian Lowland, located upwind (Fig. 1b), are the main potential dust sources for the NIGP: 1) Modern observations indicate that the present dust in northern Iran is mainly carried by the near-surface north-westerlies and north-easterlies from the neighboring Karakum Desert and arid Caspian Lowland^42^. 2) The Pleistocene loess in the NIGP and its surrounding regions mainly covers the north-facing slopes, suggesting that the ancient dust was mainly transported from the north. 3) The grain size and thickness of the last glacial loess decrease gradually from the NIGP in the north to the northern foothills of the Alborz Mountains in the south^23^,^42^, suggesting an overall southward transport pathway. 4) The geochemical signature of the lower Pleistocene LPS is similar to that of the overlying upper Pleistocene loess and modern loess (Fig. 2f), suggesting similar dust sources.

The lower Pleistocene loess in the NIGP, with a basal age of ~2.4 Ma, is the oldest loess found so far in western ACA. Its characteristics are consistent with those of the lower Pleistocene loess reported from south Tajikistan in central ACA^19^. Two early Pleistocene loess records from western and central ACA indicate that there was widespread loess accumulation in ACA during the early Pleistocene. Accumulation of loess is commonly associated with the occurrence of dust storms at the studied sites^1^,^10^,^11^,^12^,^19^, and the widespread distribution of loess is evidence that dust storms have become frequent in ACA at least since the early Pleistocene.

The LPS in the mid-latitudes of Asia are indicative of a semi-arid environment^1^. The transition from shallow marine sediments to loess deposits at ~2.4 Ma in the NIGP documents a dramatic change in the early Pleistocene from a region with a humid, marine-influenced climate^43^ to a semi-arid climate. Fundamentally, the formation of widespread, thick loess deposits requires a substantial source area arid enough to generate aeolian particles, sufficient wind energy to transport the dust, and suitable geomorphological conditions for preserving the deposited dust^44^. Therefore, the accumulation of loess deposits in the NIGP at ~2.4 Ma suggests that an arid environment had formed in the source regions, such as the Karakum Desert and the Caspian Lowland, during the early Pleistocene. This remarkable early Pleistocene aridification in western ACA is broadly consistent with the onset of aridification in central ACA^19^, the expansion of the Taklimakan Desert in northwestern China^34^,^45^, and intensified aridification in northeastern China^3^,^10^,^46^. This suggests that a semi-arid to arid environment developed within a large region of mid-latitude Asia in the early Pleistocene.

The NIGP is located in the westernmost part of ACA (Fig. 1a) and the loess deposits in the region can be regarded as an important link between the central Asian and European loess belts. Systematic palaeo-climatic reconstruction based on the LPS in the Danube Basin in southeastern Europe suggest a progressive increase in the climatic continentality of the southeastern European lowlands since the middle Pleistocene^47^,^48^. The early Pleistocene LPS in northern Iran is younger than the Tajik loess and the Chinese loess to the east, and is older than the southeastern European loess to the west. This suggests that a westward extension of aridity from central Asia to southeastern Europe occurred during the transition from the early to the middle Pleistocene.

Alternations of loess layers and palaeosols in the NIGP document long-term palaeoclimatic changes in western ACA, which are reflected by colour variations. Cross-spectral analysis demonstrates that the amplitudes of the peaks in spectral density of the redness record from the AB1 section closely match those of the marine oxygen isotope record at the 41-ka periodicity (Fig. S6), documenting the occurrence of loess-palaeosol alternations in the NIGP on an orbital time scale. Based on the magnetostratigraphic time scale, variations in redness in the AB1 section are both correlative with the redness record of the Lingtai section^31^ in the CLP, and clearly in-phase with the LR04 stacked benthic δ^18^O curve^32^ during the early Pleistocene (Fig. 4, and see *SI* Text for more information). That is, higher redness values (hence higher temperature and humidity) correspond to lower δ^18^O values (less terrestrial ice and higher temperatures), and *vice versa* (Fig. 4). A recent study of the middle-upper Pleistocene LPS in northern Iran indicated that the topmost loess layers accumulated after ~66 ka, while the underlying palaeosol complex developed since ~127 ka^23^, correlating with the last glacial loess (L1) and the last inter-glacial palaeosol (S1) from the NIGP^49^, respectively. Together with the middle Pleistocene loess records from south Tajikistan^20^,^22^, we assume that the orbital-scale palaeoclimatic changes in ACA overall were in-phase with those of monsoonal Asia during the Pleistocene.

The foregoing indicates that the orbital-scale palaeoclimatic changes in ACA were closely linked to the growth and decay of the northern hemisphere ice sheets. There are two possible causes of this relationship. First, the expansion of the ice sheets in the northern hemisphere resulted in cooling of the North Atlantic Ocean and enhanced continentality in the Asian interior^50^. This would have reduced moisture transport to the continental interior and thus increased its aridity^17^, resulting in the accumulation of loess deposits in ACA. An alternative interpretation is that the expansion of the ice sheets intensified the Mongolian-Siberian high pressure system which forced the southward migration of the zonal Westerlies^17^,^18^, which would have resulted in the enhanced incursion of cold air masses derived from high latitudes of Eurasia and reduced the influx of moisture from the Atlantic Ocean^21^.

## Methods

### Dating

Palaeomagnetic and biostratigraphic dating were used to establish a chronological framework for the red-coloured strata of the AB1 section. In addition, high resolution measurements of redness (a*) were correlated with the redness record on the CLP^31^ and the orbitally-tuned LR04 stacked benthic δ^18^O (%) record^32^ to test and further refine the primary chronology.

### Magnetostratigraphy

255 oriented block samples were collected from the red-colored strata from the most complete part of the AB1 section, at a sampling interval of ca. 10 cm. In the laboratory, the oriented samples were cut into cubes with 2-cm sides. Inside a magnetically shielded (<200 nT) laboratory, each specimen was progressively demagnetized in a MMTD80 thermal demagnetizer using up to 14 temperature steps with intervals of 100 °C up to 300 °C, 50 °C from 300 to 500 °C, and 30 °C from 500 to 670 °C. After each demagnetization step, the natural remanent magnetization (NRM) was measured using a 2G 760R Superconducting Rock Magnetometer. All of the palaeomagnetic data were analyzed using the PMag31b2 software developed by Craig Jones^51^.

### Sedimentological methods

Sedimentary facies were recognized in the field based primarily on colour, composition, texture, fossils, and sedimentary structures^33^. Subsequently, scanning electron microscope (SEM), micromorphology, geochemical and grain size analyses were performed to provide independent evidence for the interpretation of the sediment origin. 510 bulk samples were collected from the red-coloured strata from the most complete AB1 section, at a 5-cm sampling interval. More than 40 bulk samples were collected from the AB2 section and the KB section, and 50 surface samples and bulk samples were collected from the overlying upper Pleistocene loess successions for comparison.

### SEM observations

20 representative samples were selected and treated with 10% H_2_O_2_ and 10% HCL to remove organic matter and carbonates, respectively, prior to examining the grain morphology using a Hitachi S4800 SEM.

### Micromorphological analysis

Undisturbed samples were collected from each soil horizon. Air dried, and thin sections of about 60 and 40 cm^2^ prepared using standard techniques^52^. Micromorphological descriptions were made according to ref. 52.

### Geochemical analysis

40 representative samples from the red-coloured strata and 2 samples from the overlying upper Pleistocene loess succession from the AB1 section were selected for geochemical analysis. The samples were treated with 1 mol/l acetic acid (HAc) for 12 hours to remove the pedogenic carbonate fraction without significantly affecting the silicates or iron oxides^53^. After rinsing, the samples were oven-dried at 100 °C for 12 h. The concentrations of 32 major, trace and rare earth elements/oxides (Cl, S, P, As, Ba, Ce, Co, Cr, Cu, Ga, Hf, La, Mn, Nb, Nd, Ni, Pb, Rb, Sr, Th, Ti, Tl, V, Y, Zn, Zr, Fe_2_O_3_, SiO_2_, Al2O_3_, CaO, Na_2_O, K_2_O) were determined using a PANalytical PW2403/00 X-ray fluorescence spectrometer.

### Grain-size analysis

Grain size was measured using a Malvern Mastersizer 2000 laser grain-size analyzer following the pre-treatment procedures described in ref. 54. 1) 3–4 g samples were treated with 10 ml of 30% H_2_O_2_ to remove organic matter; 2) carbonates were removed with 10 ml of 10% HCL; 3) acidic ions were removed by adding 100 ml of distilled water, and leaving the sample suspension for 12 h before rinsing; 4) the sample residues were dispersed with 10 ml of 0.5 N (NaPO_3_)_6_ followed by treatment with an ultrasonic vibrator before measurement.

### Colour measurements

Each air-dried sample was gently crushed, taking care not affect the grain size, and then measured using a Konica-Minolta CM-700 colour meter. Colour reflectance was defined by the CIE L*a*b* color space. L* represents lightness and ranges from 0 to 100; a* indicates the red/green component, with green as negative values and red as positive values; b* indicates the blue/yellow component, with blue as negative values and yellow as positive values.

### Magnetic susceptibility measurements

Low-frequency (0.47 kHz) magnetic susceptibility, χ_LF_ , was measured using a Bartington Instruments meter and MS2B sensor. All of the measurements were made in the Key Laboratory of Western China’s Environmental systems, Lanzhou University.

## Additional Information

**How to cite this article**: Wang, X. *et al*. Early Pleistocene climate in western arid central Asia inferred from loess-palaeosol sequences. *Sci. Rep.* **6**, 20560; doi: 10.1038/srep20560 (2016).

## Supplementary Material

Supplementary Information

## References

[b1] Liu T. S. Loess and the Environment 303–320 (China Ocean Press, 1985).

[b2] Zhang X. Y. . Sources of Asian dust and role of climate change versus desertification in Asian dust emission. Geophys Res. Lett. 30, doi: 10.1029/2003GL0182062272 (2003).

[b3] Rea D. K., Leinen M. & Janecek T. R. Geologic approach to the long-term history of atmospheric circulation. Science 227, 721–725 (1985). 17796713 10.1126/science.227.4688.721

[b4] Bory A. J. M., Biscaye P. E. & Grousset F. E. Two distinct seasonal Asian source regions for mineral dust deposited in Greenland (North GRIP). Geophys Res. Lett. 30, doi: 10.1029/2002GL016446 (2003).

[b5] Harrison S. P., Kohfeld K. E., Roelandt C. & Claquin T. The role of dust in climate changes today, at the last glacial maximum and in the future. Earth-Sci. Rev. 54, 43–80 (2001).

[b6] Chen F. H. . Holocene moisture evolution in arid central Asia and its out-of-phase relationship with Asian monsoon history. Quaternary Sci. Rev. 27, 351–364 (2008).

[b7] Huang W., Chen J. H., Zhang X. J., Feng S. & Chen F. H. Definition of the core zone of the “westerlies-dominated climatic regime”, and its controlling factors during the instrumental period. Sci. China Earth Sci. 58, 676–684 (2015).

[b8] Chen F. H. . Moisture changes over the last millennium in arid central Asia: a review, synthesis and comparison with monsoon region. Quaternary Sci. Rev. 29, 1055–1068 (2010).

[b9] Chen J. H. . Hydroclimatic changes in China and surroundings during the Medieval Climate Anomaly and Little Ice Age: spatial patterns and possible mechanisms. Quaternary Sci. Rev. 107, 98–111 (2015).

[b10] An Z. S., Kutzbach J. E., Prell W. L. & Porter S. C. Evolution of Asian monsoons and phased uplift of the Himalaya-Tibetan plateau since Late Miocene times. Nature 411, 62–66 (2001). 11333976 10.1038/35075035

[b11] Guo Z. T. . Onset of Asian desertification by 22 Myr ago inferred from loess deposits in China. Nature 416, 159–163 (2002). 11894089 10.1038/416159a

[b12] Heller F. & Liu T. S. Magnetostratigraphical dating of loess deposits in China. Nature 300, 431–433 (1982).

[b13] Sun D. H., Shaw J., An Z. S., Cheng M. Y. & Yue L. P. Magnetostratigraphy and paleoclimatic interpretation of a continuous 7.2 Ma Late Cenozoic eolian sediments from the Chinese Loess Plateau. Geophys Res. Lett. 25, 85–88 (1998).

[b14] Ding Z. L. . Pedostratigraphy and paleomagnetism of a 7.0 Ma eolian loess-red clay sequence at Lingtai, Loess Plateau, north-central China and the implications for paleomonsoon evolution. Palaeogeogr.Palaeocl. 52, 49–66 (1999).

[b15] Zhang Y. B. . Cenozoic record of aeolian sediment accumulation and aridification from Lanzhou, China, driven by Tibetan Plateau uplift and global climate. Global Planet.Change. 120, 1–15 (2014).

[b16] Kukla G. & An Z. S. Loess stratigraphy in Central China. Palaeogeogr. Palaeocl. 72, 203–225 (1989).

[b17] Porter S. C. & An Z. S. Correlation between climate events in the North Atlantic and China during the last glaciation. Nature 375, 305–308 (1995).

[b18] Ding Z. L. . Ice-volume forcing of East Asian winter monsoon variations in the past 800,000 years. Quaternary Res. 44, 149–159 (1995).

[b19] Dodonov A. Loess of central Asia. Geo. Journal 24, 185–194 (1991).

[b20] Ding Z. L. . The loess record in southern Tajikistan and correlation with Chinese loess. Earth Planet. Sc. Lett. 200, 387–400 (2002).

[b21] Machalett B. . Aeolian dust dynamics in central Asia during the Pleistocene: Driven by the long-term migration, seasonality, and permanency of the Asiatic polar front. Geochem.Geophy.Geosy. 9, doi: 10.1029/2007GC001938 (2008).

[b22] Yang S. L., Ding F. & Ding Z. L. Pleistocene chemical weathering history of Asian arid and semi-arid regions recorded in loess deposits of China and Tajikistan. Geochim. Cosmochim. Ac. 70, 1695–1709 (2006).

[b23] Frechen M., Kehl M., Rolf C., Sarvati R. & Skowronek A. Loess chronology of the Caspian lowland in northern Iran. Quatern. Int. 198, 220–233 (2009).

[b24] Kehl M. Quaternary Loesses, Loess-like Sediments, Soils and Climate Change in Iran 81-85 (Gebrüder Borntraeger Verlagsbuchhandlung, 2010).

[b25] Pye K. Aeolian Dust and Dust Deposits 214–215 (Academic Press Londres, 1987).

[b26] Retallack G. J. Colour Guide to Paleosols 96–104 (John Wiley & Sons. Ltd., 1997).

[b27] Jahn B. M., Gallet S. & Han J. M. Geochemistry of the Xining, Xifeng and Jixian sections, Loess Plateau of China: eolian dust provenance and paleosol evolution during the last 140 ka. Chem. Geol. 178, 71–94 (2001).

[b28] Vandenberghe J. Grain size of fine-grained windblown sediment: A powerful proxy for process identification. Earth-Sci. Rev. 121, 18–30 (2013).

[b29] Sun D. H. . Grain-size distribution function of polymodal sediments in hydraulic and aeolian environments and numerical partitioning of the sedimentary components. Sediment. Geol. 152, 263–277 (2002).

[b30] Gradstein F. M., Ogg J. G. & Smith A. G. A Geologic Time Scale 2004 409–452 (Cambridge Univ. Pr., 2004).

[b31] Wang F. . Quantitative reconstruction of paleo-temperature and paleo-precipitation of Lingtai profile in Loess Plateau during the past 7 Ma. J. of Earth Environment 3, 1022–1035 (2012). (In Chinese with English Abstract)

[b32] Lisiecki L. E. & Raymo M. E. A Plio-Pleistocene stack of 57 globally distributed benthic δ^18^O records. Paleoceanography 20, 522–533 (2005).

[b33] Reading H. Sedimentary Environments: Processes, Facies, and Stratigraphy 48–49 (Wiley-Blackwell, 1996).

[b34] Sun D. H. . Palaeomagnetic and palaeoenvironmental study of two parallel sections of late Cenozoic strata in the central Taklimakan Desert: Implications for the desertification of the Tarim Basin. Palaeogeogr.Palaeocl. 300, 1–10 (2011).

[b35] Chen J. . Variations in chemical compositions of the eolian dust in Chinese Loess Plateau over the past 2.5 Ma and chemical weathering in the Asian inland. Sci. China Ser. D-Earth Sci. 44, 403–413 (2001).

[b36] Buggle B., Glaser B., Hambach U., Gerasimenko N. & Marković S. An evaluation of geochemical weathering indices in loess–paleosol studies. Quatern. Int. 240, 12–21 (2011).

[b37] Dasch E. J. Strontium isotopes in weathering profiles, deep-sea sediments and sedimentary rocks. Geochim.Cosmochim. Ac. 33, 1521–1552 (1969).

[b38] Chen F. H., Bloemendal J., Wang J. M., Li J. J. & Oldfield F. High-resolution multi-proxy climate records from Chinese loess: evidence for rapid climatic changes over the last 75 kyr. Palaeogeogr.Palaeocl. 130, 323–335 (1997).

[b39] Vandenberghe J., Mücher H. J., Roebroeks W. & Gemke D. Lithostratigraphy and palaeoenvironment of the Pleistocene deposits at Maastricht-Belvédère, southern Limburg, The Netherlands. Mededelingen Rijks Geologische Dienst 39, 7–29 (1985).

[b40] Dearing J. Environmental Magnetic Susceptibility 5–7 (Chi. Publ., 1994).

[b41] Muhs D. R. Loess deposits, origins and properties. In Encyclopedia of Quaternary Science (ed Elias S. A.) 1405–1418 (Elsevier, 2007).

[b42] Okhravi R. & Amini A. Characteristics and provenance of the loess deposits of the Gharatikan watershed in northeast Iran. Global Planet.Change. 28, 11–22 (2001).

[b43] Popov S. V. . Lithological-Paleogeographic Maps of Parathys, 10 Maps Late Eocene to Pliocene 40–41 (Courier Forschungsinstitut Senckenberg, 2004).

[b44] Pye K. The nature, origin and accumulation of loess. Quaternary Sci. Rev. 14, 653–667 (1995).

[b45] Wang X. . A high-resolution multi-proxy record of late Cenozoic environment change from central Taklimakan Desert, China. Clim. Past. 9, 2731–2739 (2013).

[b46] Sun Y. B. & An Z. S., Late Pliocene-Pleistocene changes in mass accumulation rates of eolian deposits on the central Chinese Loess Plateau. J. Geophys. Res.: Atmos. 110, doi: 10.1029/2005JD006064 (2005).

[b47] Buggle B. . The progressive evolution of a continental climate in southeast-central European lowlands during the Middle Pleistocene recorded in loess paleosol sequences. Geology 41, 771–774 (2013).

[b48] Marković S. B. . Danube loess stratigraphy-Towards a pan-European loess stratigraphic model. Earth-Sci. Rev. 148, 228–258 (2015).

[b49] Lu Y. C., Wang X. L. & Wintle A. G. A new OSL chronology for dust accumulation in the last 130,000 yr for the Chinese Loess Plateau. Quaternary Res. 67, 152–160 (2007).

[b50] Clark P. U., Alley R. B. & Pollard D. Northern Hemisphere Ice-Sheet Influences on Global Climate Change. Science 286, 1104–1111 (1999).

[b51] Jones C. H. User-driven integrated software lives: “Paleomag” paleomagnetics analysis on the Macintosh. Comput. Geosci. 28, 1145–1151 (2002).

[b52] Stoops G. Guidelines for the Analysis and Description of Soil and Regolith Thin Sections 8–177 (SSSA, 2003).

[b53] Chen J., Wang H. T. & Lu H. Y. Behaviors of REE and other trace elements during pedological weathering-evidence from chemical leaching of loess and paleosol from the Luochuan section in central China. Acta Geol. Sin. 70, 61–72 (1996).

[b54] Lu H. Y. & An Z. S. Experimental study on the influence of different pretreatment procedures on the particle-size measurement of loess sediments. Chin. Sci. Bull. 42, 2535–2538 (1997).

[b55] Maman S., Blumberg D. G., Tsoar H., Mamedov B. & Porat N. The Central Asian ergs: A study by remote sensing and geographic information systems. Aeolian Res. 3, 353–366 (2011).

[b56] Hollingsworth J., Jackson J., Walker R., Reza Gheitanchi M. & JavadBolourchi M. Strike-slip faulting, rotation, and along-strike elongation in the KopehDagh mountains, NE Iran. Geophys. J. Int. 166, 1161–1177 (2006).

[b57] Gerakis A. & Baer B. A computer program for soil textural classification. Soil Sci. Soc. Am. J. 63, 807–808 (1999).

[b58] Paillard D., Labeyrie L. & Yiou P. Macintosh program performs time-series analysis. Eos, Transactions American Geophysical Union 77, 379–379 (1996).

