# Supplementary material for: Early Pleistocene climate in western arid central Asia inferred from loess-palaeosol sequences
Source: Sci Rep. 2016 Feb 3;6:20560. doi: 10.1038/srep20560 (PMC4738352; doi:10.1038/srep20560)
Supplement: Supplementary Information [file srep20560-s1.pdf]

Supplementary Information

Early Pleistocene climate in western arid central Asia inferred from loess-palaeosol sequences

Xin Wang[1*], Haitao Wei[1], Mehdi Taheri[2], Farhad Khormali[2], Guzel Danukalova[3], Fahu Chen[1*]

Supplementary Text

Text S1: Geological setting

The Iranian north Glostan Province (INGP) in northern Iran is located in the westernmost part of arid central Asia (ACA) (Fig. 1a). It is flanked by the Kopet Dag Range to the north, by the Alborz Mountains to the south, and by the Caspian Sea to the west (Fig. 1b). Tectonically, the INGP is located in the western part of the Kopet-Dag fold belt (Fig. 1b), where a series of faults developed in response to the accelerated collision between the Arabian and Turan plates from the Pliocene to the present[S1-S3]. During most of Cenozoic time, the INGP and its surrounding regions were periodically occupied by a large inland sea (Fig. 1a), Paratethys, which covered large areas of the mid-latitudes of Eurasia[4] and which had a prolonged impact on the climate of Asia[S4-S7].

At present, the INGP is characterized by a semi-arid climate with a strong seasonality in precipitation. The mean annual temperature is about 17 °C, and the mean annual precipitation is less than 350 mm with more than 85% falling during the winter and spring months, largely associated with the southward migration of the Westerlies during the boreal winter[S8]. The near-surface wind regime is dominated by north-westerly to north-easterly winds driven by the pressure differences between the Caspian Sea Basin and the Central Iranian highlands[S9]. As a result, large amounts of dust from the Karakum Desert and the arid Caspian Lowland are transported downwind to the INGP[S10].

The reported middle-upper Pleistocene loess-palaeosol profiles in northern Iran are distributed on the INGP (Agh Band section) and along the northern foothills of the Alborz mountains between the cities of Sari and Minodasht[S9-S12] (Fig. 1b). These loess profiles consist of an alternation of homogenous dull, yellowish-coloured (2.5 Y 5/3) loess layers and well-developed brown (7.5 YR 4/3) palaeosol beds, deposited in glacial and interglacial periods, respectively[S9, S11, S12]. Infrared optically stimulated luminescence (IRSL) dating suggested that the basal age of the loess deposits in the Agh Band section from the INGP was around 145±14 ka[S12], representing the oldest loess known so far in the INGP.

Red-coloured non-marine sediments are widely distributed in the INGP (Fig. 1b, Fig. S1). They unconformably underlie the upper Pleistocene loess successions[S12] and conformably overlie the late Cenozoic limestone sequences that include abundant mollusc shells (Fig. 2a). The sedimentology of three well-exposed sections (Fig. S1), namely AB1 (37°41'20" N, 55°9'30" E), AB2 (37°38'10" N, 55°12'53" E), and KB (37°36'1" N, 55°25'19" E), was investigated and the most complete AB1 section was selected for magnetostratigraphic study.

Fig. S1

Text S2: Chronological framework

Biostratigraphic analysis of the underlying limestone sequences

Mollusc species collected from the underlying limestone sequences include *Aktschagylia subcaspia* (Andrus.), *A.* cf. *ossoskovi* (Andrus.), *A.* cf. *karabugasica* (Andrus.), *Cerastoderma* cf. *dombra pseudoedule* (Andrus.), *C.* cf. *dombra dombra* (Andrus.); *C.* cf. *altum* (Tschelt.), Cardiidae *(Cerastoderma), Miricardium* sp., Mactridae *(Aktschagylia)* (Fig. S2). These species are indicative of shallow marine environments and confirm the Akchagylian age of the deposits[S13-S14]. The basal age of the Akchagylian stage is 3.2 Ma based on bio-magnetostratigraphic dating with constraints provided by fission track dating of volcanic ashes. However, the age of the top is poorly constrained, with estimates ranging from ~2.4 to 1.8 Ma[S15]. In any case, the limestone and the overlying red beds in the AB1 section must be younger than 3.2 Ma.

Fig. S2

Magnetostratigrapy of the reddish loess-palaeosol sequence in the AB1 section

Magnetic hysteresis loops of representative samples from the AB1 section are narrow and are closed at around 400 mT (Fig. S3), suggesting that the magnetic carriers are mainly magnetically 'soft', with a low coercivity[S16]. The thermomagnetic curves exhibit a major drop in magnetization near 580 °C, followed by a progressive decrease until 680 °C (Fig. S3), indicating the presence of both magnetite and hematite[S16].

Fig. S3

On orthogonal vector diagrams[S17] most of the samples exhibit two magnetic components (Fig. S4). The low-stability component is generally unblocked at low temperatures ranging from room temperature to 200 °C and its direction is broadly consistent with the present day field; it is thus interpreted as a secondary viscous remanent magnetization (VRM). The second component has relatively stable directions and decays linearly towards the origin (Fig. S4). This high-stability component is interpreted as the characteristic remanent magnetization (ChRM), the mean direction of which was calculated using principal component analysis (PCA) of a least-squares fit[S18]. Generally, the samples with maximum angular deviations (MAD) larger than 15° were considered to fail the significance test in directional analysis and were rejected for further analysis. Parallel samples were measured in these cases and they also did not pass the significance test. In total, 167 reliable ChRM directions were obtained from the 187 measured data (89.3 %). After tilt correction, the mean normal and reverse polarity directions are: D/I = 357.0°/50.5° (K=3.58, N=16), and D/I = 192.0°/−51.9° (K = 22.53, N = 151) respectively. The ChRM directions passed the reversal test for palaeomagnetic stability[S19], suggesting that the ChRMs obtained are the primary NRMs.

Fig. S4

The palaeomagnetic polarity of the red-coloured strata in the AB1 section is defined by the virtual geomagnetic poles (VGP) calculated from the ChRM declination and inclination data (Fig. S5). The polarity record consists of two normal and two reversal intervals, designated N1, R1, N2, and R2, from the top to the bottom of the section. In addition, a stable and reliable normal point was obtained at 10.7 m, which may record a geomagnetic excursion (Fig. S5).

Fig. S5

With the aforementioned biostratigraphic age constraints, the magnetostratigraphy for the red beds in the AB1 section is easily correlated to the Matuyama reversal polarity zone (C1r.1r to C2r.2r) of the geomagnetic polarity time scale[S20]. There are two possibilities for additional correlations. The first possibility is that N1 and N2 correlate to the Olduvai event (C2n) and the Reunion event (C2n.1n), and that the normal event at 10.7 m correlates to the geomagnetic excursion occurring at 2.19 Ma (Fig. S5). Based on the resulting age model, the sediment redness record exhibits a similar pattern of variation to the redness record of the Lingtai section from the CLP[S21], and the LR04 stacked benthic $\delta^{18}O$ record[S22] (Fig. 4). An alternative option is

that N1 and N2 correlate to the Jaramillo event (C1r.1n) and the Cobb Mountain event (C1r.2n), respectively (Fig. S5). However, this correlation is probably incorrect for the following reasons: 1) Using this age model most of the non-marine sediments were deposited during an interval equivalent to the middle Apsheron transgression of the Caspian Sea, and the estimated sea level was about 100 m higher than at present[S23]. Since the studied sections are located in a former marine environment (Fig. 1a), deposition of non-marine sediments at this time is in conflict with the regional geological history. 2) The normal event at 10.7 m cannot be correlated to any known geomagnetic excursions[S20]. 3) The redness record exhibits no correlation with the deep-sea $\delta^{18}O$ record[S22], conflicting with the middle Pleistocene loess records from mid-latitude Asia[S24-S25].

Based on the most likely magnetostratigraphic correlation, the sediment accumulation rates within the R1 and N2 intervals are ca. 3.03 cm/kyr and 2.29 cm/kyr, respectively. By using the sediment accumulation rate from the neighboring stratum, the basal and top ages of the red beds from the AB1 section are inferred to be ~2.383 and ~1.815 Ma, respectively.

Comparison of the redness (a) record of the AB1 section and the stacked benthic $\delta^{18}O$ (‰) record*

We used the redness (a*) record as a high resolution proxy climate index for correlation to the LR04 stacked benthic $\delta^{18}O$ (‰) record[S22] for the following reasons: 1) The attitudes of the red-coloured strata in the red beds are the same and the

alternations of palaeosol and loess-like horizons are highly comparable among the AB1, AB2 and KB sections (Fig. S1). This suggests that the red-coloured sediments were deposited continuously without significant hiatuses, at least on an orbital time scale. 2) Colour variations are the most readily visualized property of the red-coloured sediments in the studied sections (Fig. 2). Both strongly-developed and weakly-developed palaeosols have higher redness (a*) values than the loess-like sediments, suggesting that redness can be used as a high resolution index for stratigraphic correlations. 3) Numerous studies have demonstrated that variations in the redness of loess-palaeosol sequences are a reliable proxy for moisture and/or temperature history in mid-latitude of Asia[S24-S27]. 4) The reported middle-upper Pleistocene loess records from northern Iran[S11-S12] and southern Tajikistan[S24] in ACA indicate that the dull yellowish-coloured loess layers (lower a* values) were deposited in glacial periods, whereas the brown palaeosol horizons (higher a* values) developed during interglacials, yielding a direct genetic linkage between Northern Hemisphere ice volume and sedimentary colours in mid-latitude Asia on orbital time-scales since the middle Pleistocene[S24-S25, S28-29].

A time series of redness (a*) of the AB1 section was established by linearly interpolating between the ages of geomagnetic polarity boundaries. Cross-spectral analysis demonstrates that the amplitudes of the peaks in spectral density of redness closely match those of the marine oxygen isotope record at the 41 ka periodicity (Fig. S6). To facilitate correlation, the 41-kyr periodicity components of the redness record

from the AB1 section, the redness record from the Lingtai Section[S21] in monsoonal Asia, and the LR04 stacked benthic $\delta^{18}O$ records[S22], were extracted by band-pass filtering using the computer program described in supplementary ref. 30.

Fig. S6

The well-constrained 'Reunion event' occurring at 2.128-2.148 Ma is used as a 'marker layer' (dark grey bar in Fig. 4) for stratigraphic correlation. It is apparent that a weakly-developed palaeosol (higher redness) formed during an interglacial period (lower $\delta^{18}O$), which is consistent with the loess records from the CLP[S32]. The corresponding peaks in redness in both the INGP and CLP are slightly lagged relative to the peak in the $\delta^{18}O$ record (Fig. 4). These lags may be attributed to different post-depositional remanent magnetization lock-in depths in loess and marine sediments and/or to bioturbation within the surface mixed layer[S32]. Based on the primary palaeomagnetic time scale, variations of redness from the INGP and CLP yield similar patterns, and exhibit an in-phase relationship with global ice volume during the early Pleistocene. That is, higher redness (higher temperature and humidity) corresponds to lower $\delta^{18}O$ (less ice and higher temperature), and *vice versa* (Fig. 4). It should be noted that the redness time scales for the AB1 and Lingtai sections[S21] were obtained by linearly interpolating between the ages of geomagnetic polarity boundaries. This method leads to dating errors within polarities, which may result in

the differences in redness between the two sections during certain intervals (e.g. 2.3-2.4 Ma). According to the correlation of marker layers (Fig. 4, dark grey bar) and middle-late Pleistocene records from ACA and monsoonal Asia[S24], we believe these 'shifts' arise from uncertainties in palaeomagnetic dating rather than from an out-of phase relationship between ACA and monsoonal Asia.

Based on the stratigraphic correlations, the lower Pleistocene loess in the AB1 section is correlated to MIS 64-93[S22]. The basal and top ages of the red-colored loess in the AB1 section are thereby refined to ~2.39 Ma and ~1.81 Ma, respectively.

Supplementary Figures

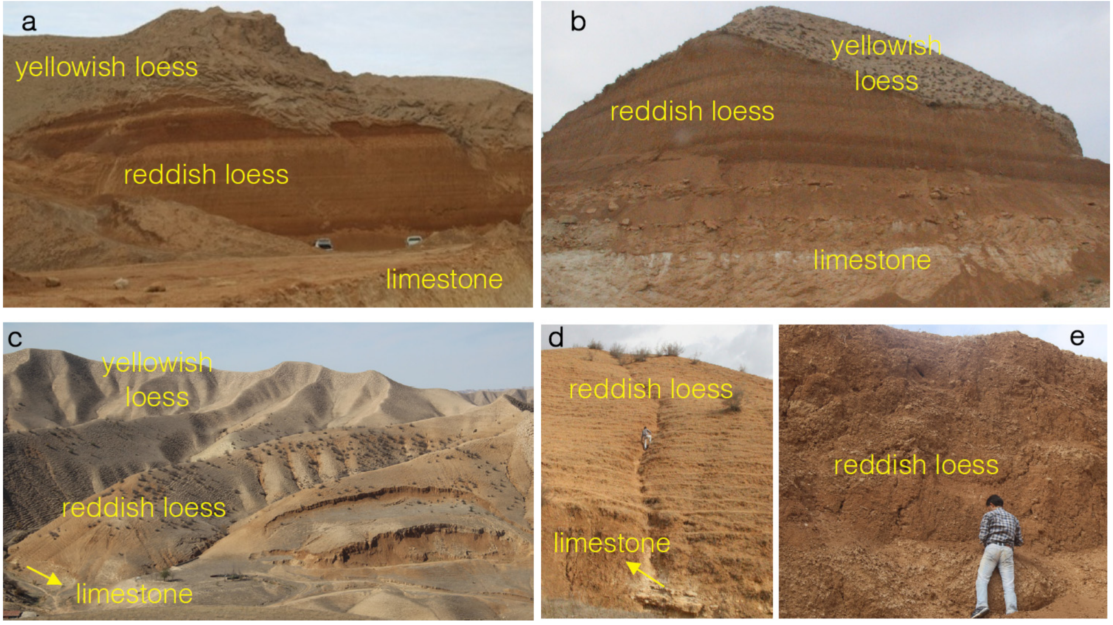

Figure S1. Photograph showing the reddish-coloured loess underlying the upper Pleistocene loess in the INGP. a) AB1 section; b) AB2 section; c- e) KB section.

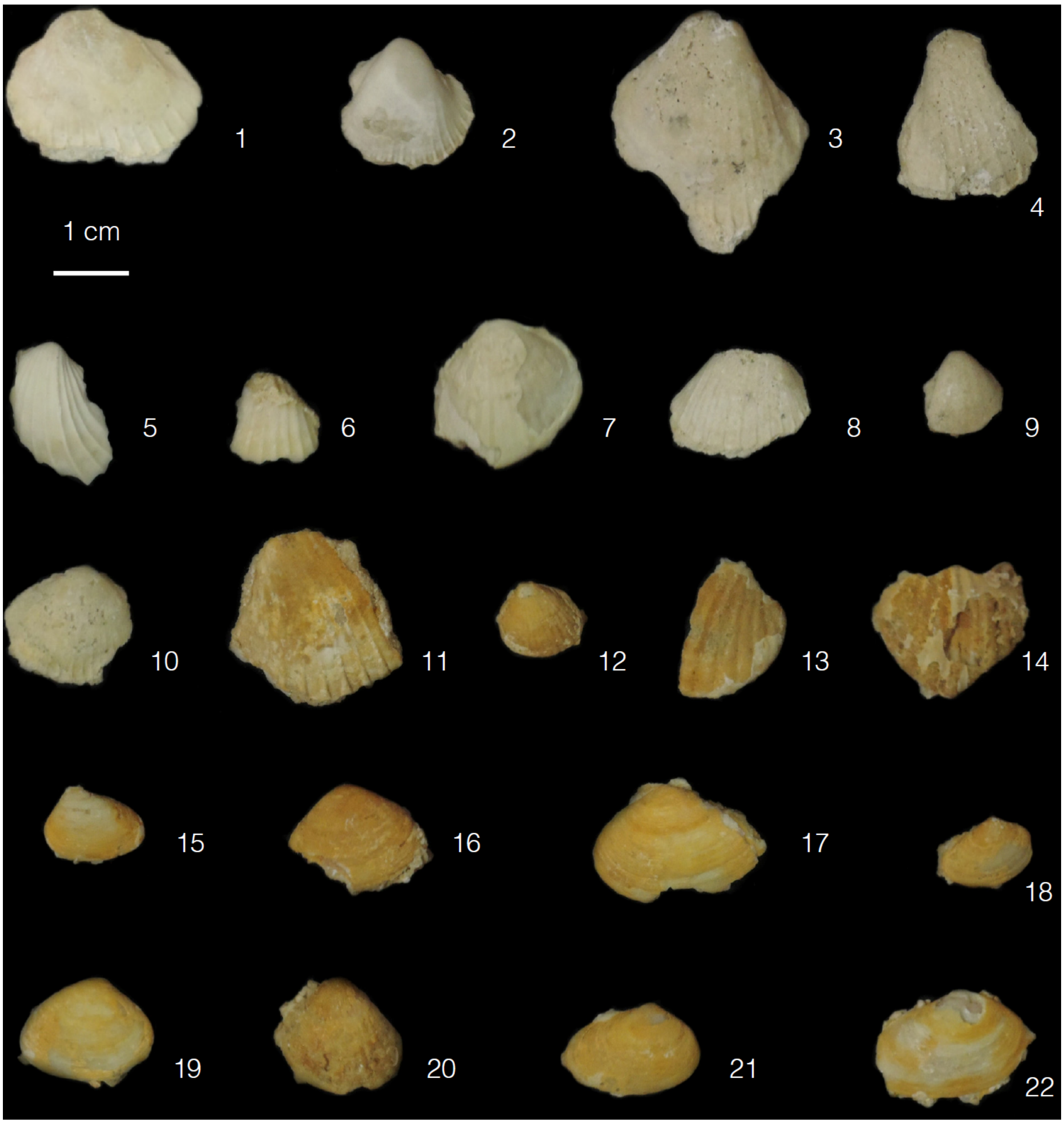

Figure S2 The most significant mollusc species identified from the limestone sequences. 1, *Cerastoderma* cf. *dombra pseudoedule* (Andrus.), left valve; 2, *Cerastoderma* cf. *dombra dombra* (Andrus.), right valve; 3-4, *Cerastoderma* cf. *altum* (Tschelt.), right valve; 5-6, *Miricardium* sp., fragments of the left (5) and right (6) valves; 7-15, Cardiidae *(Cerastoderma)*, left and right valves; 16, *Aktschagylia subcaspia* (Andrus.), left valve; 17-18, Mactridae *(Aktschagylia),* fragments of the left and right valves; 19, *Aktschagylia subcaspia* (Andrus.), left valve; 20, *Aktschagylia* cf. *karabugasica* (Andrus.), right valve; 21, *Aktschagylia* cf. *ossoskovi* (Andrus.), right valve; 22, *Aktschagylia subcaspia* (Andrus.), left valve; 23, *Aktschagylia subcaspia* (Andrus.) or *Aktschagylia* cf. *karabugasica* (Andrus.), right valve; 1-23, outside shell view. All samples are represented by inside cores of the Bivalve shells.

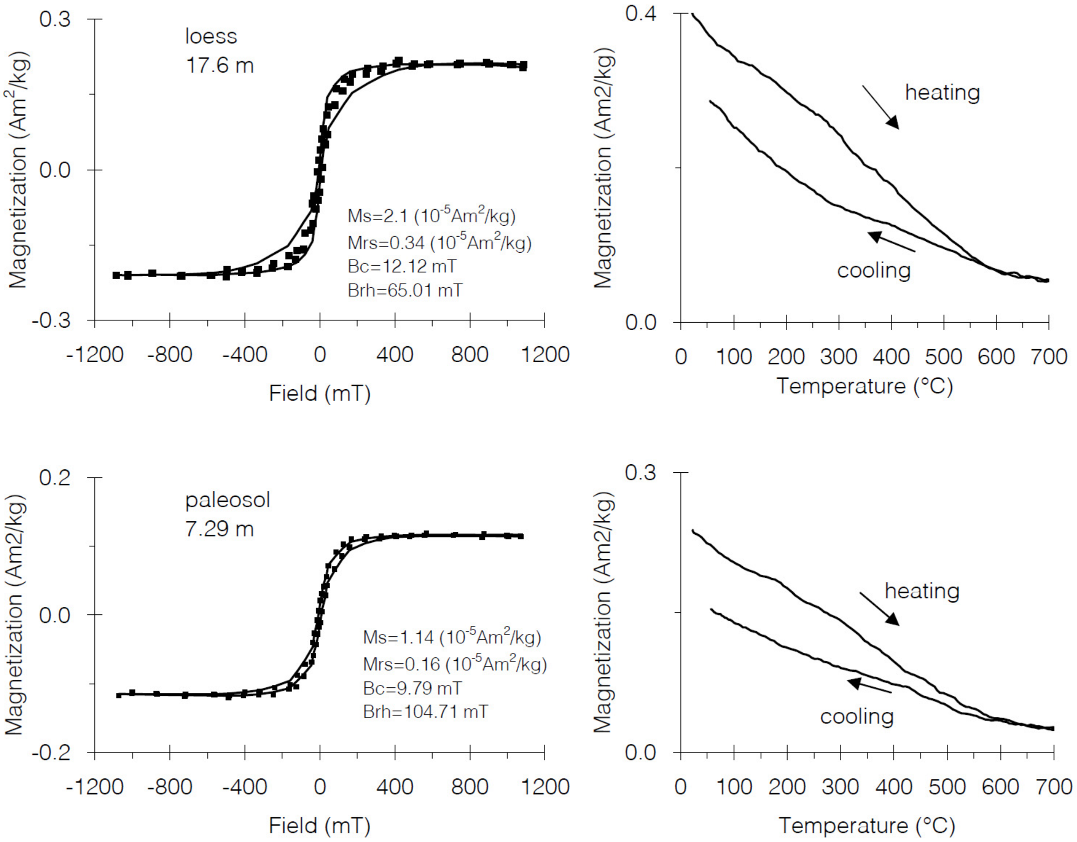

Figure S3. Hysteresis loops and thermomagnetic curves for representative samples from the red-coloured strata of the AB1 section. The paramagnetic susceptibility was calculated from the slope of the magnetization curve between ~800 mT and 1000 mT, and used to subtract the paramagnetic contribution to the loops. The magnetizing field used for the thermomagnetic experiments was 360 mT.

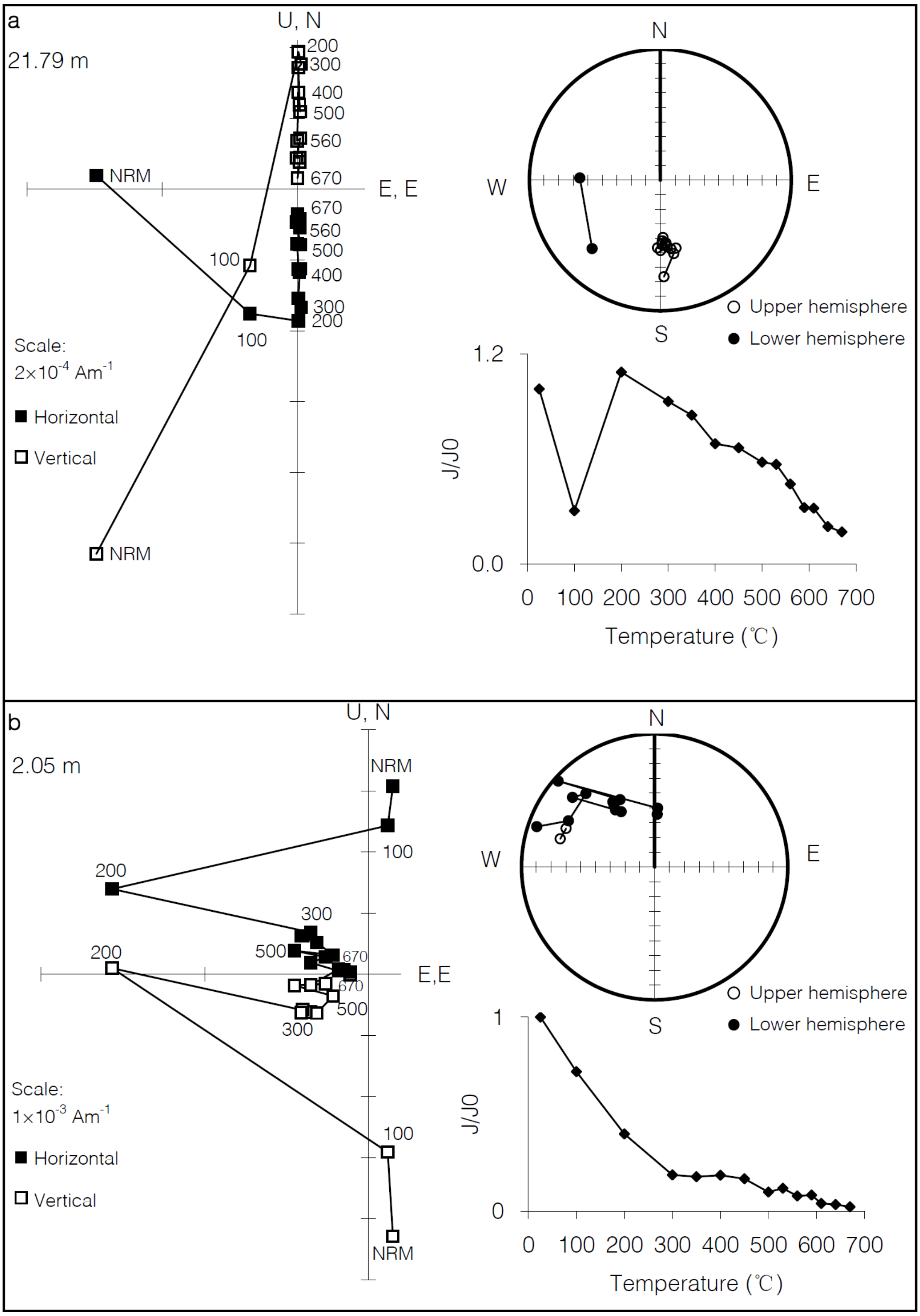

Figure S4. Plots showing demagnetization of orthogonal components, equal-area projection of the demagnetization data and normalized NRM intensity versus thermal demagnetization temperature for representative samples from the AB1 section. Numbers adjacent to the NRM directions in the vector component diagram indicate the demagnetization level (in °C).

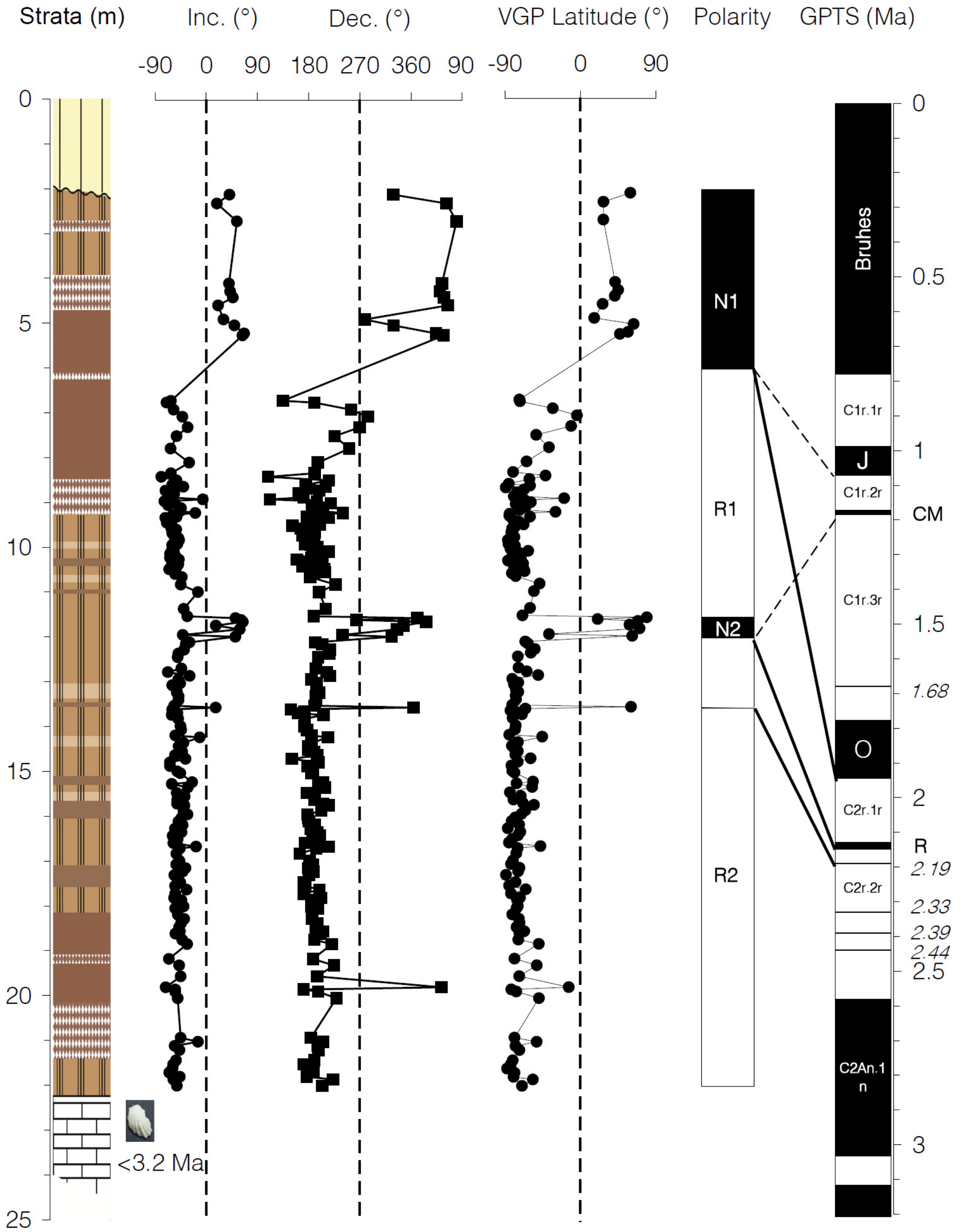

Figure S5. Lithology, inclination (Inc.), declination (Dec.), VGP latitude and inferred polarity of the AB1 section together with its correlation with GPTS 2004[S20]. Abbreviations in the GPTS are: CM, Cobb Mountain event; and R, Reunion event. The numbers in italics beyond the GPTS 2004 indicate the numerical age of geomagnetic excursions that occurred during the Matuyama reversed interval.

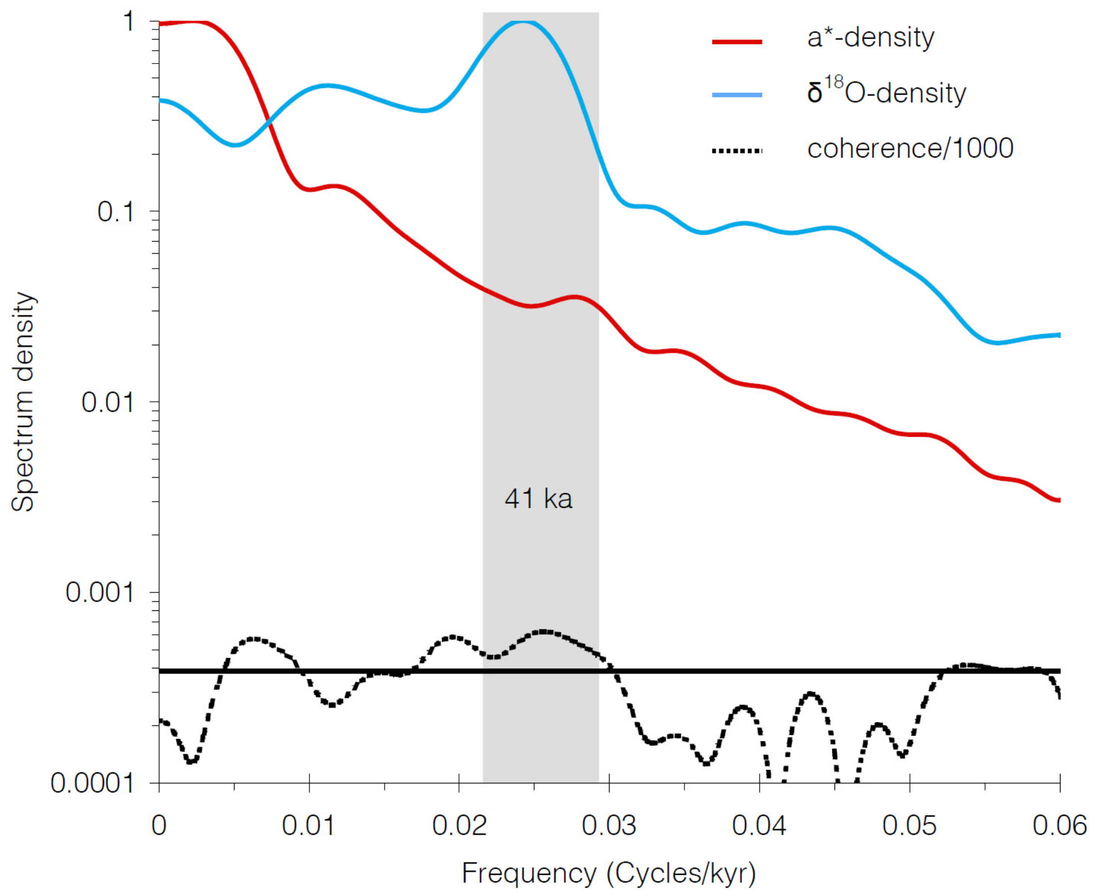

Figure S6. Cross spectral analysis of the redness record from the AB1 section, using the primary palaeomagnetic time scale, and a stacked $\delta^{18}O$ record[S22] over the time interval 1.8-2.5 Ma. Spectral density is normalized and plotted on a log scale. The solid straight line denotes the 80% significance level of coherence. Shaded bars indicate the dominant spectral peaks for these records. Note the high coherence within the 41 kyr periodicity band.

Supplementary References

S1. Hollingsworth, J., Jackson, J., Walker, R., Reza Gheitanchi, M. & Javad Bolourchi, M. Strike-slip faulting, rotation, and along-strike elongation in the Kopeh Dagh mountains, NE Iran. *Geophys. J. Int.* **166,** 1161-1177 (2006).

S2. Javidfakhr, B., Bellier, O., Shabanian, E., Ahmadian, S. & Saidi A. Plio-Quaternary tectonic regime changes in the transition zone between Alborz and Kopeh Dagh mountain ranges (NE Iran). *Tectonophysics* **506,** 86-108 (2011).

S3. Bretis, B., Grasemann, B. & Conradi, F.. An active fault zone in the Western Kopeh Dagh (Iran). *Austrian J. Earth Sci.* **105,** 95-107 (2012).

S4. Popov, S. V. *et al*. *Lithological-Paleogeographic Maps of Parathys, 10 Maps Late Eocene to Pliocene* 40-41 (Courier Forschungsinstitut Senckenberg, 2004).

S5. Bosboom, R. *et al*. Timing, cause and impact of the late Eocene stepwise sea retreat from the Tarim Basin (west China). *Palaeogeogr. Palaeocl.* **403,** 101-118 (2014).

S6. Wang, X. *et al*. Cenozoic paleo-environmental evolution of the Pamir–Tien Shan convergence zone. *J. Asian Earth Sci.* **80,** 84-100 (2014).

S7. Carrapa, B. *et al*. Tectono-climatic implications of Eocene Paratethys regression in the Tajik basin of central Asia. *Earth Planet. Sc. Lett.* **424,** 168-178 (2015).

S8. Ballato, P. *et al*. Middle to late Miocene Middle Eastern climate from stable oxygen and carbon isotope data, southern Alborz Mountains, N Iran. *Earth Planet. Sc. Lett.* **300,** 125-138 (2010).

S9. Kehl, M. *Quaternary Loesses, Loess-like Sediments, Soils and Climate Change in Iran* 81-85 (Gebrüder Borntraeger Verlagsbuchhandlung, 2010).

S10. Okhravi, R. & Amini, A. Characteristics and provenance of the loess deposits of the Gharatikan watershed in northeast Iran. *Global Planet. Change.* **28,** 11-22 (2001).

S11. Kehl, M., Sarvati, R., Ahmadi, H., Frechen, M. & Skowronek, A. Loess paleosol-sequences along a climatic gradient in Northern Iran. *Eiszeitalter und Gegenwart* **55,** 149-173 (2006).

S12. Frechen, M., Kehl, M., Rolf, C., Sarvati, R. & Skowronek, A. Loess chronology of the Caspian lowland in northern Iran. *Quatern. Int.* **198,** 220-233 (2009).

S13. Danukalova, G. Bivalves and Aktschagylian stratigraphy. *Trudy Paleontologicheskogo Instituta Rossia Akademii Nauk* **265,** 1-132 (1996).

S14. Yakchemovitcht, V., Danukalova, G. & Yakovlev, A. Molluscs and small mammals from Pliocene deposits of the Middle Volga region, Russia. *Mededelingen Nitherlands Instituut voor Toegepaste–Geowetenschappen TNO* **60,** 175-416 (1998).

S15. Van Baak, C. G. C. *et al*. A magnetostratigraphic time frame for Plio-Pleistocene transgressions in the South Caspian Basin, Azerbaijan. *Global Planet. Change.* **103,** 119-134 (2013).

S16. Tauxe, L. *Essentials of Paleomagnetism* 65-84 (University of California Press, 2009).

S17. Zijderveld, J. D. A. AC demagnetization of rocks: analysis of results. in *Methods In Paleomagnetism.* (eds Collison, D. W., Runcorn, S.K. & Creer, K.M.) 254-286 (Elsevier, 1967).
S18. Kirschvink, J. L. The least-squares line and plane and the analysis of palaeomagnetic data. *Geophys. J. Roy. Astron. Soc.* **62,** 699-718 (1980).
S19. McFadden, P. L. & McElhinny, M. W. Classification of the reversal test in palaeomagnetism. *Geophys. J. Int.* **103,** 725-729 (1990).
S20. Gradstein, F. M., Ogg, J. G. & Smith, A.G. *A Geologic Time Scale 2004* 409-452 (Cambridge Univ. Pr., 2004).
S21. Wang, F. *et al.* Quantitative reconstruction of paleo-temperature and paleo-precipitation of Lingtai profile in Loess Plateau during the past 7 Ma. *J. of Earth Environment* **3,** 791-791 (2012). (In Chinese with English Abstract)
S22. Lisiecki, L. E. & Raymo, M. E. A Plio-Pleistocene stack of 57 globally distributed benthic $\delta^{18}O$ records. *Paleoceanography* **20,** 522–533 (2005).
S23. Forte, A. M. & Cowgill, E. Late Cenozoic base-level variations of the Caspian Sea: A review of its history and proposed driving mechanisms. *Palaeogeogr. Palaeocl.* **386,** 392-407 (2013).
S24. Ding, Z. L. *et al.* The loess record in southern Tajikistan and correlation with Chinese loess. *Earth Planet. Sc. Lett.* **200,** 387-400 (2002).
S25. Yang, S. L., Ding, F. & Ding, Z. L. Pleistocene chemical weathering history of Asian arid and semi-arid regions recorded in loess deposits of China and Tajikistan. *Geochim. Cosmochim. Ac.* **70,** 1695-1709 (2006).
S26. Sun, D. H. *et al.* Palaeomagnetic and palaeoenvironmental study of two parallel sections of late Cenozoic strata in the central Taklimakan Desert: Implications for the desertification of the Tarim Basin. *Palaeogeogr. Palaeocl.* **300,** 1-10 (2011).
S27. Sun, Y. B., He, L., Liang, L. J. & An Z. S. Changing color of Chinese loess: Geochemical constraint and paleoclimatic significance. *J. Asian Earth Sci.* **40,** 1131-1138 (2011).
S28. Forster, T. & Heller F. Loess deposits from the Tajik depression (Central Asia): Magnetic properties and paleoclimate. *Earth Planet. Sc. Lett.* **128,** 501-512 (1994).
S29. Frechen, M. & Dodonov A. E. Loess chronology of the Middle and Upper Pleistocene in Tadjikistan. *Geol Rundsch* **87,** 2-20 (1998).
S30. Paillard, D., Labeyrie, L. & Yiou P. Macintosh program performs time - series analysis. *Eos, Transactions American Geophysical Union* **77,** 379-379 (1996).
S31. Sun, D. H., Shaw, J., An, Z. S., Cheng, M. Y. & Yue, L. P. Magnetostratigraphy and paleoclimatic interpretation of a continuous 7.2 Ma Late Cenozoic eolian sediments from the Chinese Loess Plateau. *Geophys Res. Lett.* **25,** 85-88 (1998).
S32. Liu, Q. S., Roberts, A. P., Rohling, E. J., Zhu, R. X. & Sun, Y. B. Post-depositional remanent magnetization lock-in and the location of the Matuyama–Brunhes geomagnetic reversal boundary in marine and Chinese loess

sequences. *Earth Planet. Sc. Lett.* 275, 102-110 (2008).
